# World blindness and visual impairment: despite many successes, the problem is growing

**Published:** 2018-02-08

**Authors:** Peter Ackland, Serge Resnikoff, Rupert Bourne

**Affiliations:** 1 CEO: International Agency for the Prevention of Blindness, London, UK.; 2 Chair, International Coalition for Trachoma Control (ICTC), Geneva, Switzerland.; 3 Consultant Ophthalmic Surgeon: Department of Ophthalmology, Hinchingbrooke Hospital, Huntingdon, UK.

**Over the last 30 years, there has been a reduction in the proportion of people with visual impairment and blindness worldwide. However, growing and ageing populations mean that the challenge of eliminating avoidable blindness is now bigger than ever before.**

We have come a long way on the journey towards the global elimination of avoidable blindness and visual impairment over the last three decades. Thanks to the work of the Vision Loss Expert Group, it is now possible to say what has been achieved and what remains to be done.

The group has very recently published detailed estimates of the prevalence of global blindness and visual impairment – for the past, present and future – at global, regional and country level.^1^ These estimates were derived from a detailed analysis of 288 population surveys conducted in 98 countries from 1980 to mid-2014. Although the estimates mainly concentrate on distance vision loss, data regarding near vision loss (presbyopia) are also included.

The wealth of information the group has produced has been summarised in the International Agency for the Prevention of Blindness (IAPB) Vision Atlas. The Vision Atlas includes a series of maps where you can find the latest estimates for your country at the click of a mouse **http://atlas.iapb.org/gvd-maps**

## Overall trends and patterns

**Figure 1:**
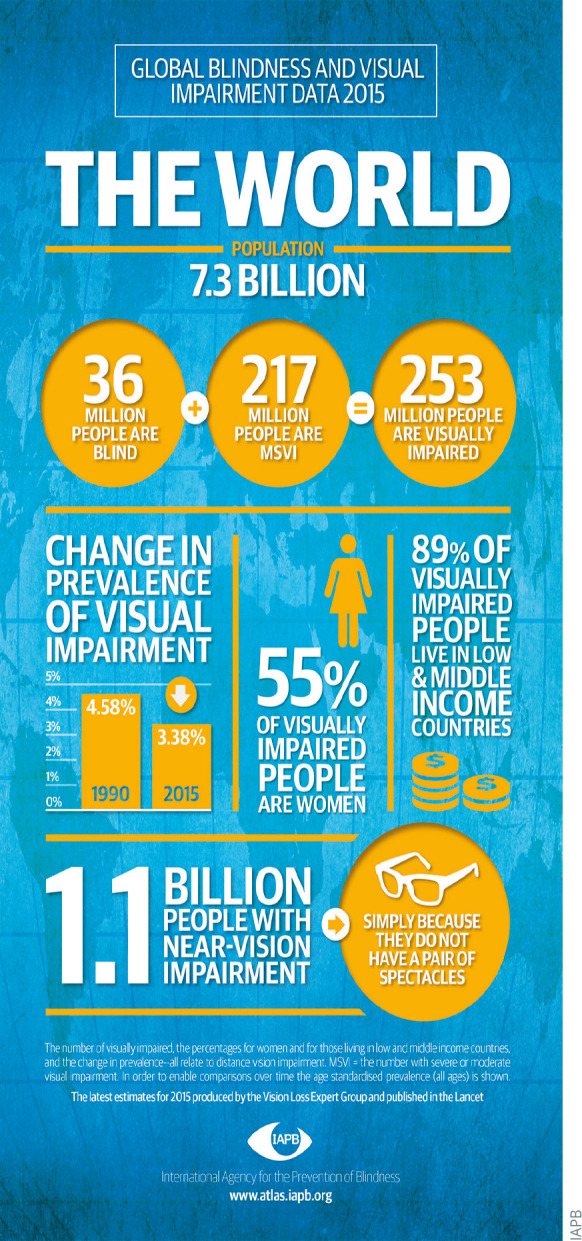
Headline figures from the Vision Loss Expert Group's latest data

The headline figures from the group's latest global estimates are summarised in Figure 1.

In 2015, there were an estimated 253 million people with visual impairment worldwide. Of these, 36 million were blind and a further 217 million had moderate to severe visual impairment (MSVI). The prevalence of people that have distance visual impairment is 3.44%, of whom 0.49% are blind and 2.95% have MSVI. A further 1.1 billion people are estimated to have functional presbyopia.

**Figure:**
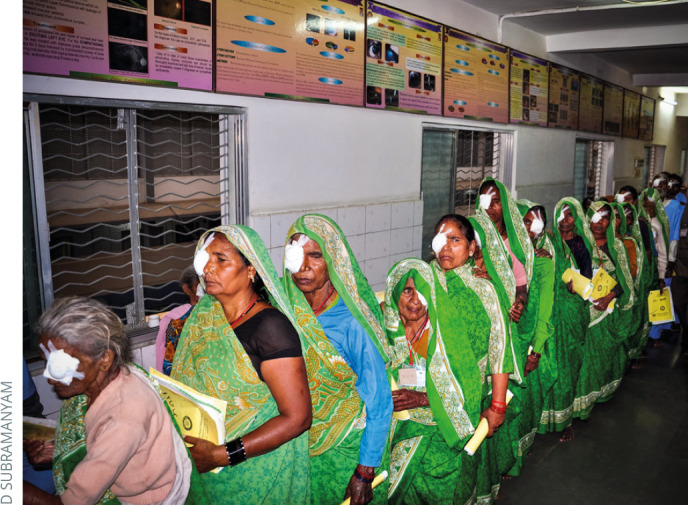
It is important that women are able to access eye care services. INDIA

### Age

**Figure 2:**
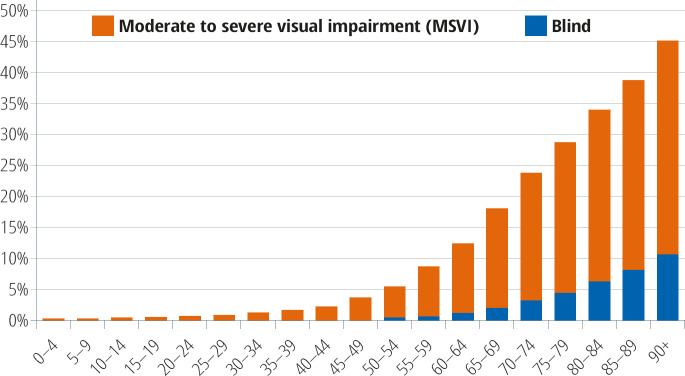
The global prevalence of blindness and moderate to severe visual impairment (MSVI) in women*

The risk of most eye conditions increases with age; consequently, the prevalence of blindness and MSVI is much greater in older age groups. Figure 2 shows this for women (a very similar result is seen for men). Of the 253 million visually impaired people worldwide, 80% are aged 50 years or older.

**Figure 3:**
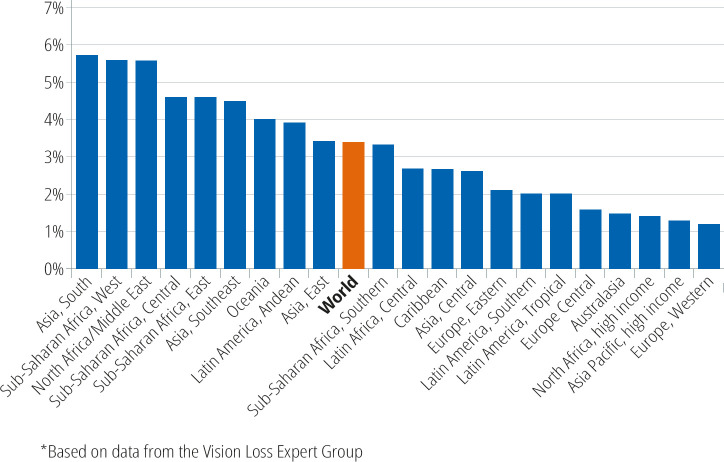
Age-standardised prevalence of visual impairment across the 21 regions*

*Based on data from the Vision Loss Expert Group

### Gender

Of the 253 million people in the world who are visually impaired, 55% are women (139 million). A number of factors contribute to this gender imbalance, including the longer life expectancy of women compared with that of men, which means that there are more women in those age groups associated with a higher risk of developing a sight-threatening eye condition (Figure 2). In addition, women are at greater risk of developing certain eye conditions. In some countries, women suffer disadvantages in terms of access to eye health services. This is due to multiple socio-economic and cultural factors.

## Regional data

The group used the 21 regions demarcated by the Global Burden of Disease study (**www.thelancet.com/gbd**) to disaggregate the global data and prepare regional estimates. These 21 regions cluster countries according to their physical location but also other factors, including their socio-economic status.

### Socio-economic status

89% of visually impaired people live in low- and middle-income countries. Three Asian regions are home to 62% of the people in the world with visual impairment, even though they are home to only 51% of the world's population: South Asia (73 million), East Asia (59 million) and South East Asia (24 million). At the other end of the scale, the five high-income regions account for 14% of the world's population but only 11% of people with visual impairment.

### Age profile

Comparing regions is not straightforward if one just looks at the overall numbers or prevalence; this is due to the differences in the age profile in each region; i.e. some populations may have a larger proportion of older people and fewer children compared to others. A technique called ‘age standardisation’ makes it possible to compare populations with different age profiles to each other and look at changes over time.

The age-standardised prevalence of visual impairment across the 21 regions is shown in Figure 3. The prevalence in poorer regions of the world is more than four times that seen in the high-income regions.

The group has also published a second paper^2^ that looks at the causes of visual impairment. Table 1 summarises the estimates of the causes of blindness, moderate to severe visual impairment, and for blindness and visual impairment combined. The data are for 2015 and are given both in terms of absolute numbers and as a percentage.

## Changes over time

The group has produced global estimates stretching back to 1990 and have also looked into the future to produce estimates for 2020 to 2050; the results are summarised in Table 2 (opposite). At first glance, the gradual increase in the absolute number of people who are blind or have MSVI from 1990 to 2015 may seem disappointing. However, over this 25-year period, two very important demographic changes have occurred, both of which would have been expected to give rise to a much greater increase in the absolute number of visually impaired people:

1. The global population increased by 38%: from 5.3 billion in 1990 to 7.3 billion in 2015.
2. The world population aged and the total population over 50 years old almost doubled: from 878 million in 1990 to 1,640 million in 2015.

**Table 1:** Vision Loss Expert Group estimates of the causes of visual impairment in 2015

Cause	Blind		Moderate to severe visual impairment		All visual impairment	
	<3/60 to no light perception (NLP)		<6/18–3/60		<6/18-NLP	
	No. millions	%	No. millions	%	No. millions	%
Cataract	12.6	35	52.6	24	65.2	26
Uncorrected refractive error	7.4	21	116.3	54	123.7	49
Glaucomas	3	8	4	2	7	3
Age-related macular degeneration	2	5	8.4	4	10.4	4
Corneal opacity	1.3	4	2.9	1	4.2	2
Trachoma	0.4	1	1.6	1	2	1
Diabetic retinopathy	0.4	1	2.6	1	3	1
All other causes	8.9	25	28.2	13	37.1	14
**Total**	**36**	**100**	**216.6**	**100**	**252.6**	**100**

Allowing for these two major changes, there is in fact an underlying decline in the global age-standardised prevalence of blindness (all ages): it has reduced from 4.58% in 1990 to 3.38% in 2015. A number of factors – including a decline in poverty levels, a reduction of the incidence of certain conditions or a later onset of these conditions, improved public health measures and eye health service development – have all contributed to this encouraging progress.

**Table 2:** Vision Loss Expert Group estimates of the global number of people who are blind or have moderate to severe visual impairment, 1990 to 2050

Year	Global number affected, all ages (millions)	
	Blindness	Moderate to severe visual impairment
1990	31	160
2000	32	176
2010	34	199
2015	36	217
2020	39	237
2030	55	330
2040	80	451
2050	115	588

**Table 3:** Vision Loss Expert Group estimates for future population growth and ageing^3^

	2015	2050
Global population	7.3bn	9.7bn
> 60 years old	0.9bn	2.1bn
> 80 years old	125m	434m

## The future

But what is likely to happen in future? United Nations data^3^, summarised in Table 3, informs us that the global population was 7.3 billion in 2015. This is predicted to rise to 7.8 billion by 2020 and to 9.7 billion by 2050.

The growing population is also going to age at a much faster rate than seen in previous years. In 2015, there were 901 million people over the age of 60 (12% of the global population). By 2050, the number of people over the age of 60 is predicted to increase to 2.1 billion (22% of the population).

An even greater relative increase in the numbers of people aged ≥80 is expected; the current estimate of 125 million in 2015 is expected to increase more than threefold by 2050: to 434 million. As observed in Figure 2, the prevalence of visual impairment increases rapidly with age. By age 60, around 1 in 9 people will be either blind or have MSVI. By age 80, the ratio increases considerably: around 1 in 3 people will be either blind or have MSVI.

The combination of a growing and an ageing population will result in a massive increase in the number of people who are blind or have MSVI. Two other factors that also present a major risk for the future are the dramatic increase currently being seen in all parts of the world in the number of people with diabetes (which can cause diabetic retinopathy, a potentially blinding condition) and those with high myopia.

Overall, there may be some 703 million people who are blind or have MSVI by the year 2050 (as shown in Table 2). A massive investment in eye health services, along with protection from out-of-pocket payments for the poorest sectors of society, is needed to ensure universal access to eye health for all and avert a future human and societal catastrophe.
